# Positron Emission Tomography for the Response Evaluation following Treatment with Chemotherapy in Patients Affected by Colorectal Liver Metastases: A Selected Review

**DOI:** 10.1155/2015/706808

**Published:** 2015-05-11

**Authors:** Alberto Zaniboni, Giordano Savelli, Claudio Pizzocaro, Pietro Basile, Valentina Massetti

**Affiliations:** ^1^Medical Oncology, Fondazione Poliambulanza, Via Bissolati 57, 25124 Brescia, Italy; ^2^Nuclear Medicine Department, Fondazione Poliambulanza, Brescia, Italy

## Abstract

The aim of the present paper is to review the scientific literature concerning the usefulness of ^18^F-FDG PET/CT in the evaluation of response to chemotherapy in patients affected by liver metastases from colorectal cancer. *Material and Methods*. Studies were identified by searching PubMed electronic databases. Both prospective and retrospective studies were included. Information regarding the figure of merit of PET for the evaluation of therapy response was extracted and analyzed. *Results*. Existing data suggests that ^18^F-FDG PET/CT may have an outstanding role in evaluating the response. The sensitivity of PET in detecting therapy response seems to be greater than conventional imaging (CT and MRI). PET/CT response is strictly related to better overall survival and progression-free survival. *Conclusions*. PET/CT is more than a promising technique to assess the response to chemotherapy in colorectal and liver metastases. However, to be fully validated, this examination needs further studies by recruiting more patients.

## 1. Introduction

^18^F-PET/CT is a well-established imaging modality in oncology, widely used to stage, restage, and follow up several malignancies, including colorectal cancer (CRC) [1–3]. In the past few years, there was a rising attention for ^18^F-PET/CT use in the evaluation of response to therapy of liver metastases from CRC. The recent marketing of expensive biologic and molecular targeted drugs has made the early evaluation of their effectiveness even more stringent. Indeed, the final mechanism of action of these compounds is more cytostatic than cytolitic. The therapeutic outcome of cytostatic drugs results in the halt of tumor growth. The lack of a sharp lowering of the neoplastic dimension has become a real challenge for conventional radiology assessment of response to therapy. Indeed, Computed Tomography (CT) and, in a lesser degree, Magnetic Resonance Imaging (MR) evaluation are traditionally based on a reduction in the main diameter of the tumor.

^18^F-FDG PET/CT is a hybrid technique, which associates the molecular imaging PET with CT. In spite of the examination's result, which resembles a particular CT scan, the major strength of this technique originates from its molecular part, that is, the possibility to evaluate the total amount of tumor metabolism through its consumption of radioactive glucose. The specific power of  ^18^F-FDG PET/CT to measure tumors' metabolism, and therefore its induced therapy alteration, makes it a theoretically ideal marker of treatment responsiveness.

Here we present a selected review of the scientific literature concerning the usefulness of  ^18^F-FDG PET/CT in the evaluation of response to systemic therapy in patients affected by liver metastases from colorectal cancer (CRCLM).

## 2. Computed Tomography

Computed Tomography (CT) is still the first choice method to evaluate the response of CRLM both in routine clinical practice and in clinical trials. The use of CT scan to evaluate the responsiveness of a neoplasm to a certain antineoplastic agent is based on the assumption that if the treatment is effective, its cytostatic/cytotoxic action will eventually induce a lowering in neoplastic mass, which is measurable by conventional radiologic equipment. Following this statement, many scientific organizations devoted to cancer treatment developed criteria aimed to define a generally accepted base to measure the response in cancer treatment [4–6].

RECIST is an international standard criteria based on a simplification of former methods (WHO, ECOG). The prerequisite for RECIST criteria evaluation is based on the presence of quantifiable disease; that is, it is mandatory that at least one lesion be measurable. These organizations offer a simplified, conservative extraction of imaging data for wide application in clinical trials. They presume that linear measures are an adequate substitute for 2D methods. RECIST records four response categories, that is, (a) complete response (CR, disappearance of all target lesions), (b) partial response PR = 30% decrease in the sum of the longest diameter of target lesions, (c) progressive disease (PD = 20% increase in the sum of the longest diameter of target lesions), and (d) stable disease (SD = small changes that do not meet the above criteria).

CT evaluation in all its multiform applications has gained an extensive use due to its reproducibility and widespread distribution. However, the geometrical mechanism at the base of morphologic imaging response is forcibly subsequent to previous molecular, biochemical, and, ultimately, metabolic changes. This characteristic has a medium to low impact on the assessment of cytotoxic drugs but may result in a reduced accuracy when dealing with the new cytostatic biological class of pharmaceuticals [7–12].

Moreover, most of the studies suggest a CT evaluation after at least three cycles of chemotherapy.

Indeed, the tumor's shrinkage may be minimal even when treatment is effective, particularly with cytostatic drugs. The difficulties of CT imaging to evaluate the lack of response after one or two cycles make it impossible to redirect patients towards a more effective strategy, with obvious additional clinical and financial costs.

Along with these concerns about the chronological limits of CT, recent reports hinted at the possibility that a reduction in the density of liver parenchyma on portal venous scans after systemic therapy due to a toxic impact on liver parenchyma could result in reduced tumor-to-liver contrast and to the underestimation of real lesion size [13].

## 3. Magnetic Resonance Imaging

In contrasts to TC, which is a predominantly morphologic technique, Magnetic Resonance Imaging may include a more significant part of functional information. Magnetic resonance spectroscopy (MRS) and dynamic contrast-enhanced (DCE) and diffusion-weighted (DW) MRI may be used to evaluate molecular, biological, and, eventually, functional modifications induced by treatments. In the path toward a more personalized medicine of CRCLM treatment, DW-MRI has shown promising results as an early predictor of response in patients undergoing chemotherapy.

Some studies have shown that baseline apparent diffusion coefficient (ADC) measurements predict therapeutic benefit, with higher ADC metastases responding poorly to chemotherapy [14–16]. Early (within days) increases in ADC values have also been shown to predict a favorable response to chemotherapy in stomach, colorectal, and breast cancers [17]. To date, however, this preliminary data does not provide a strong enough evidence for the adoption of DWI as a routine examination for individualized patient management.

Another item to be clarified concerns the sensitivity of DW-MRI for metastases with different model of vascular pattern. Hypervascular metastases have shown lower ADC values compared to hypovascular metastases as in most of the cases CRLM are [18]. These preliminary data support the statement that liver metastases are not a homogenous group of lesions with uniform DW-MRI features. The reduced sensitivity of DW-MRI for CRLM may hamper the evaluation of the treatment.

## 4. ^**18**^F-FDG PET/CT

^18^F-fluorodeoxyglucose (FDG) is a glucose analog that is mostly taken up in malignant cells because of their higher glucose metabolism. Standalone ^18^F-FDG PET first, and PET/CT later on, was shown to be effective for initial staging and follow-up in oncology patients affected by most prevalent tumors (lung cancer, breast cancer, colorectal cancer, and lymphoma). These diagnostic performances warranted a strong role to ^18^F-FDG PET/CT in the diagnosis and staging work-up of many neoplasms. If we take a look to the more endorsed application of  ^18^F-FDG PET/CT, it is clear that the philosophy of PET/CT use is somehow unbalanced toward anatomic, topographic, and morphologic parameters (the location of the uptake areas, their relationship with the surrounding anatomic structures, etc.). In the first few years of its clinical use, the only ^18^F-FDG PET/CT molecular characteristics, which have been mainly utilized, were the Standard Uptake Value (SUV) in the prognostic stratification. Indeed, the higher uptake of radioactive glucose is associated with the most biologically aggressive forms of the neoplasms [19–24]. The strategy to favor the morphologic interpretation of  ^18^F-FDG PET/CT, however, risks pauperizing the ^18^F-FDG PET/CT power since it yields the underestimation of the molecular feature of the technique.

Hence, although anatomic interpretation of PET scans has been shown to be the basis of the clinical report, the development of strategies directed to gain quantitative information should allow more objective diagnosis and, above all, the comparisons between serial PET of a patient.

The rationale for the use of  ^18^F-FDG PET/CT in the evaluation of the response to systemic treatments is based on amplified glucose metabolism characteristic of neoplastic cells [25]. The mechanism of the increased glycolytic activity typical of some neoplasms, which is realized in both hypoxic [26] and normoxic conditions [27–29], is not completely understood. Probably this biologic change is the final result of a complex and multifactorial interaction between the neoplastic cell and the surrounding environment.

The theoretical construction for ^18^F-FDG PET/CT tumor's response has been extensively cleared up in the first decade of this century [30]. Basically, if we consider the results of the previous studies, a neoplasm does not become detectable until it reaches a size of 10–100 g (from 10^10^ to 10^11^ cells). This means that if standard cancer therapies have a cytolytic effect with first-order kinetics, a dose of therapy that produces a 90% reduction in tumor mass needs to be repeated 11 times to eliminate a newly diagnosed cancer. Current PET/CT systems have a spatial resolution ranging from 0.4 to 1.0 cm, corresponding to a tumor size of 0.1–0.5 to 1.0 g (10^8^-10^9^ cells). As a consequence of this, in an ideal model, the temporal window in which ^18^F-FDG PET/CT is resolute enough to monitor the lowering of the whole glucose metabolism is during the first two cycles. A negative ^18^F-FDG PET/CT after the last cycle of systemic therapy does not necessarily mean that neoplasm has been eradicated. Of course this is a theoretical model and must be considered for what it is, with all its simplifications and generalizations, but it is a good starting point to describe a biologic phenomenon. On a different note, if we consider the usefulness of PET/CT in treatment evaluation, the intimate and proportional correlation between increased neoplastic cell metabolism and growth and the raise of glucose metabolism must be taken into account. This means that the lowering of the former one, induced by treatments, should necessarily reflect in an abatement of the latter [31].

^18^F-FDG PET/CT may reach the highest outcome for CRCLM in two distinct clinical scenarios, that is, the prognostic stratification after preoperative chemotherapy and the early evaluation of systemic treatment irrespectively of the following treatments. This quite rough schematization is not an end in itself, but it follows the clinics which group patients with CRLM in three separate groups: (1) those with easily resectable disease, (2) those with borderline resectable or high recurrence risk CRLM, and (3) those with inoperable but liver limited CRLM. For the first group of patients the standard of care therapy is surgery, followed by adjuvant therapy if considered. The second group of patients should be treated with systemic neoadjuvant followed by liver surgery. The third group of patients, those with inoperable CRLM, should be offered the most effective systemic therapy with the goal to reach maximal disease response with the intention of conversion to surgical resectability with curative intent [32]. Preoperative treatment allows the monitoring of chemoresistance and identifies tumors with aggressive biology [33]. ^18^F-FDG may be used in all the cases in which an evaluation of chemotherapy effectiveness is needed and likewise in the second and third group of patients.

Surgery together with systemic chemotherapy is the only cure for patients with CRCLM [34]. Surgical techniques are quite safe but morbidity may be as high as 40% [35]. Therefore, a reliable and repeatable prognostic indicator should be able to predict 2-year overall survival (OS) and disease-free survival (DFS) [36, 37]. Metabolic response to preoperative chemotherapy is the best prognostic indicator compared with CT, prognostic scoring systems, and histological tumor regression [37, 38]. Another strong asset concerning the usefulness of  ^18^F-FDG PET/CT to stratify patient prognosis is related to the ability of molecular imaging to foresee the biologic characteristics of surely present, but still undetectable, micrometastases. The induced chemotherapy, lowering the glucose metabolism in large liver metastases, will probably reflect the same behavior of undetectable micrometastatic tumor deposits. This is possibly the main reason for the strongest relationship between metabolic response to OS and tumor regression grade [37]. In summary, metabolic response provides a measure that may be used to decide which patient affected by CRLM may have the highest gain from surgery.

The other field in which ^18^F-FDG PET/CT may play a fundamental role is the early assessment of responsiveness to systemic treatments. This is particularly true for patients treated with the novel molecular targeted drugs which have a predominantly cytostatic effect. These treatments act mainly by halting tumor growth rather than eliminating neoplastic cells. Due to their mechanism of action, therefore, molecular targeted drugs stabilize rather than kill malignant cells. Thus, despite active treatments, conventional imaging may show some changes or even an increase in tumor size, especially in the first phases of therapy, due to inflammatory changes. ^18^F-FDG PET/CT may bridge the gap between the start of cytostatic treatments effect and the response evaluation. Changes in neoplastic glucose consumption have been noticed as early as 24 hours after a dose of treatment in Gastrointestinal Stromal Tumors [39], and therefore preceding by weeks or months the lowering of anatomic parameters.

Keeping in mind the lesson of GIST, some authors evaluated the possible role of  ^18^F-FDG PET/CT in early response assessment of CRCLM with the goal to redirect nonresponding patients towards a more effective treatment. Generally, ^18^F-FDG PET/CT was carried out after 1 or 2 cycles of therapy, usually scheduled with Folfox-Folfiri plus bevacizumab [40–45]. All these studies evidenced that metabolic imaging fits better than conventional imaging and pathologic response to OS and progression-free survival (PFS). Interestingly, ^18^F-FDG PET/CT response has a stronger correlation with OS and PFS than pathological response. This is probably because the ^18^F-FDG PET/CT is able to define the biologic nature of unavoidably present, but still undetectable, metastases mirroring it from the result of the visible disease.

As ever, nuclear medicine is the vocation to give quantitative answers. This statement was true dealing with gamma cameras, but it has largely been emphasized with PET and PET/CT. Many PET/CT parameters have been studied in clinical practice. SUV represents an index for FDG uptake in tissues. This parameter was studied in the last decade of the last century [46] and subsequently validated as a marker of treatment response in women affected by breast cancer [47].

SUV is a quantification of normalized radioactivity concentration in PET images. SUV is calculated drawing a bidimensional (ROI) or tridimensional (VOI) region of interest inside the tumor lesion using software.

The measured radioactivity is then normalized to the average total radioactivity present in the body, which is hypothesized as the injected dose divided by the patient body weight (SUVbw) or the patient lean body mass (SUVlbm or SUL) or body surface area (SUVbsa). SUVmax in a VOI is the most used parameter because it is probably more reproducible due to the self-determining ROI/VOI assessment. SUVpeak is a composite measurement calculating the local average SUV within all the voxels close to the one with the highest radioactivity.

The most frequent source of error affecting SUV family measurement is ^18^F-FDG extravasation. When ^18^F-FDG PET/CT is repeated in the same patient to evaluate the outcome of a treatment it is mandatory both to inject the same activity of the radiopharmaceutical and to scan the patient at the same interval.

Other more recent methods to evaluate tumor metabolism are Total Lesion Glycolysis (TLG) [48] which allows measuring the metabolism of the whole neoplastic volume [49] and Standardized Added Metabolic Activity (SAM) [43] which has been showed to significantly split responders versus nonresponders.

In summary, ^18^F-FDG PET/CT brings with it the opportunity to measure a number of parameters, each one mirroring a specific molecular, biologic, and metabolic feature. But how have all these pieces of information been considered in clinical guidelines?

The first attempt to do so was carried out by European Organization Research and Treatment of Cancer (EORTC) in 1999 [50]. In this position paper, EORTC defined all the items connected with ^18^F-FDG PET/CT examination, that is, patient preparation, timing of scans, attenuation correction and dose of  ^18^F-FDG to be administered, methods to measure the uptake, tumor sampling, reproducibility, and definition of tumor response. With regard to the latter topic, four categories were defined: Complete Metabolic Response (CMR), Partial Metabolic Response (PMR), Stable Metabolic Disease (SMD), and Progressive Metabolic Disease (PMD). The specific characteristics for each classification are reported in Table 1. EORTC final statement in 1999 was as follows: tumor response with ^18^F-FDG PET is in its infancy. There is a requirement for larger-scale trials together with collection of reproducibility data, to assess the technique in relation to other methods of response assessment and clinical end-points. The position of EORTC is not significantly changed in the update of RECIST 1.1 which suggests ^18^F-FDG PET/CT as an adjunct to the determination of progression, and the use of these promising newer approaches (which could either add to or substitute the anatomical assessment as described in RECIST) requires appropriate and rigorous clinical validation studies.

The cornerstone of nuclear medicine therapy response evaluation could be represented by the paper of Wahl et al. [30] published in 2009. In this paper, the author compares and critically revises the morphologic and functional methods to evaluate the response of oncologic imaging, defines the technical aspects of functional imaging, and, eventually, names these criteria (Positron Emission Tomography Response Criteria In Solid Tumors, PERCIST). In PERCIST cancer response is a continuous and time-dependent variable. A tumor may be evaluated at any number of times during treatment, and glucose use may rise or fall from baseline values. Tumor SUL parameter is used and the background ^18^F-FDG activity is measured in the right hepatic lobe in a 3 cm diameter VOI. The SUL is determined for up to 5 lesions (up to 2 per organ). To date automated methods for PERCIST evaluation are widely available on the usual PET/CT systems. In PERCIST, response is assessed as a continuous variable and expressed as percentage change in SUL peak measured before and after therapy. PERCIST criteria have a great potential and promise to have a large impact of future management of neoplastic patients but still need an overall validation. Indeed, four trials are registered at National Cancer Institute to validate PERCIST in different clinical scenarios, one of which dealt with CRCLM treatment response (NCT01318447).

A recent paper has compared EORTC and PERCIST criteria in the assessment treatment with irinotecan and cetuximab in CRCLM [51].  Table 2 shows the result of the comparison of the two criteria and the correlation to OS.

The study evidenced that patients with a PR as assessed by ^18^F-FDG PET/CT have a significantly better median OS compared with those grouped as SD + PD, regardless of which criteria have been used. For all the other figures of merit, both criteria have very similar results, probably because their measurements are restricted to the most metabolically active part of the patient's tumor burden. Moreover, the lack of discrepancies provides excellent credentials to the measurement of SUV for treatment response. The authors concluded that PERCIST criterion is somehow preferable because it is more defined and less operator-dependent in nature.

## 5. Conclusion

The prognosis of patients affected by CRCLM is heavily influenced by the response to treatments. ^18^F-FDG PET/CT is a hybrid imaging method, which holds the power of both anatomic and molecular imaging. This double characteristic makes ^18^F-FDG PET/CT the ideal tool to evaluate the outcome antineoplastic therapies. The results deduced from our analysis of the scientific literature are promising and should spur the researchers towards more structured, possibly multicentric, and prospective trials.

## Figures and Tables

**Table 1:** EORTC response criteria for ^18^F-FDG PET/CT.

CMR	Complete resolution of [^18^F]-FDG uptake within the tumour volume so that it was indistinguishable from surrounding normal tissue

PMR	Reduction of a minimum of 15 ± 25% in tumour ^18^F-FDG SUV after one cycle of chemotherapy and greater than 25% after more than one treatment cycle

SMD	Increase in tumour ^18^F-FDG SUV of less than 25% or a decrease of less than 15% and no visible increase in extent of ^18^F-FDG tumour uptake (20% in the longest dimension)

PMD	Increase in ^18^F-FDG tumour SUV of greater than 25% within the tumour region defined on the baseline scan and visible increase in the extent of ^18^F-FDG tumour uptake (20% in the longest dimension) or the appearance of new ^18^F-FDG uptake in metastatic lesions

**Table 2:** Comparison of the two methods to assess the response and overall survival (OS).

	EORTC (patients)	OS (months)	PERCIST (patients)	OS (months)
CMR	0	n.a.	0	n.a.
PMR	38	14.2	34	14.5
SMD	16	6.4	20	6.9
PMD	7	12.2	7	12.2
SMD + PMD	23	7.2	27	7.9
